# NEWS AND NOTICES

**Published:** 2010-09

## Two losses for eye care

We are sad to report the deaths of Drs Moses Chirambo and Tom Little in recent months.

**Figure:**
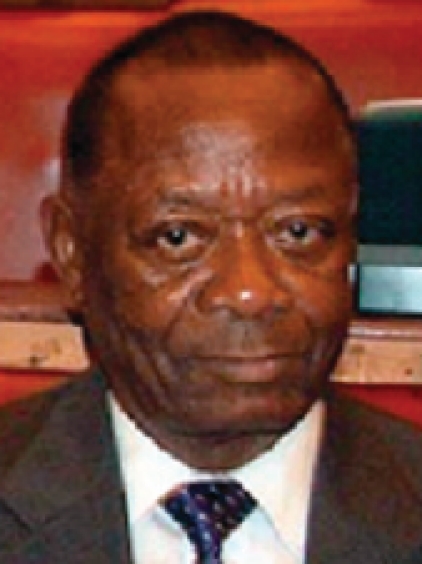


**Moses Chirambo** was Malawi's first ophthalmologist and was later appointed minister of health for his country. He passed away on August 14 during medical treatment in South Africa. Dr Chirambo worked for Sightsavers as Eye Care Programme Consultant for the East, Central, and Southern African Region; he also established and ran the Southern African Development Community (SADC) ophthalmologist course at the Malawi College of Health Sciences. He pioneered Malawi's first eye training school in the capital Lilongwe and helped establish similar programmes in Botswana, Namibia, Zimbabwe, and Zambia.

**Figure:**
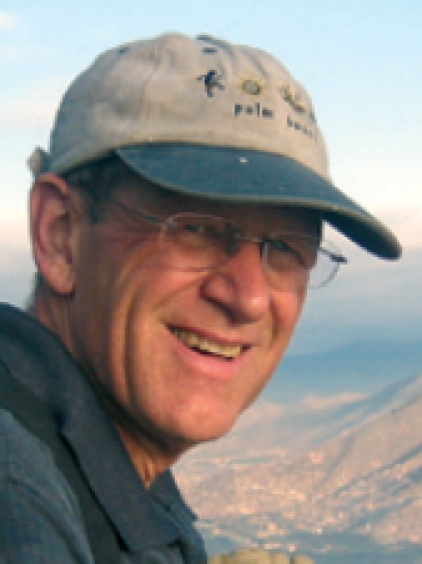


**Tom Little,** an optometrist from New York, was killed during outreach work in Nuristan province, Afghanistan, on 6 August. Dr Little had worked in eye care in Afghanistan for thirty years and supervised a network of eye hospitals around the country. At the time of his death, he was leading a team of nurses, doctors, and logistics personnel providing specialist eye treatment and health care to people in remote communities.

## Have you got news for us?

We would like to hear from you about new developments in eye care in your country or region. You can also let us know if you have meetings, courses, or events you would like to announce in the journal. Write to The Editor, International Centre for Eye Health, London School of Hygiene & Tropical Medicine, Keppel Street, London, WC1E 7HT, UK or email **editor@cehjournal.org**

## Get your own copy

Do you live in a low- or middle-income country? Did you know you can get your own, free copy of this journal, delivered **free** to your home address?

All you have to do is send your name, occupation, and postal address to Anita Shah, International Centre for Eye Health, London School of Hygiene & Tropical Medicine, Keppel Street, London, WC1E 7HT, UK, or email her on Anita.Shah@Lshtm.ac.uk

This journal is for **all health workers with eye care responsibilities,** whether you are a nurse, community health worker, medical doctor or specialist ophthalmologist. If you know of anyone else who will benefit from reading this journal, please tell them how easy it is to subscribe, or send their details directly to us.

## Erratum

The answer to our CPD question 1D (page 19 of Issue 72) should be FALSE. There is in fact a great deal that can and must be done for a blind infant by specialist instructors (specialised occupational therapists and early intervention specialists for the visually impaired) to support both the infant and the infant's family. We hope that you spotted the error and apologise for any confusion.

## Meetings

**Access Africa:** The 5th Institutional Development Programme (IDP) Africa Forum, Ghana Institute of Management and Public Administration, July 3-8, 2011. Themes include technology, access, and social and economic empowerment for people who are blind or partially sighted. For more information, email **africaforum2011@gmail.com** or write to Aubrey Webson, c/o Perkins International, 175 North Beacon Street, Watertown, MA 02472 USA.

## Courses

**Lions Aravind Institute of Community Ophthalmology (LAICO)**

LAICO offers **instrument maintenance courses** with a trainee:trainer ratio of 1:1. Four courses take place in 2011, starting on 1 February, 1 May, 1 August, and 1 November respectively. Duration: six weeks per course. Cost: US $400 (including tools). Location: LAICO, Aravind Eye Care Systems, Madurai, India. Visit **www.aravind.org/education/coursedetails.asp** for more information or write to Prof V Srinivasan, LAICO, 72, Kuruvikaran Salai, Gandhi Nagar, Madurai 625 020, Tamil Nadu, India. Email: **v.srinivasan@aravind.org**

**Kilimanjaro Centre for Community Ophthalmology (KCCO)**

All courses held in Moshi, Tanzania. For information, contact Genes Mng'anya, KCCO, Good Samaritan Foundation, P.O. Box 2254 Moshi, Tanzania, Tel +255 27 275 3547. Email: **genes@kcco.net** or visit **www.kcco.net**

**Bridging communities and eye care providers to achieve VISION 2020 in Africa:** 8-12 November 2010

**Management for VISION 2020 programme managers:** 15-26 November 2010

**Writing a research manuscript:** 29 November to 3 December 2010

**Community Eye Health Institute, South Africa**

**Certificate course in community eye health.** Aim: to provide training in the management of district VISION 2020 programmes. Start date: February 2011. Duration: 10 weeks. Cost: ZA R16,500 (approximately US $2,400) plus living expenses in Cape Town. Email **cehi@uct.ac.za** or write to Community Eye Health Institute, University of Cape Town, Private Bag 3, Rondebosch, 7700, South Africa.

**Video and photo competition**

What does the Community Eye Health Journal mean to you? Send us a photograph or short video showing how you use the journal in your daily work – whether to teach others, to refresh your knowledge, or to improve the lives of patients. The overall winner will receive Kanski's Clinical Ophthalmology, kindly donated by Elsevier (worth UK £164).

**Extended deadline**: 1 March 2011. **Patient permission:** if your photograph or video shows any patients, you must get their written permission and include this in your entry.

**Send to:** The Editor, Community Eye Health Journal, International Centre for Eye Health, London School of Hygiene and Tropical Medicine, Keppel Street, London WC1E 7HT, UK, or email editor@cehjournal.org More information: www.cehjournal.org/competition

**Figure:**
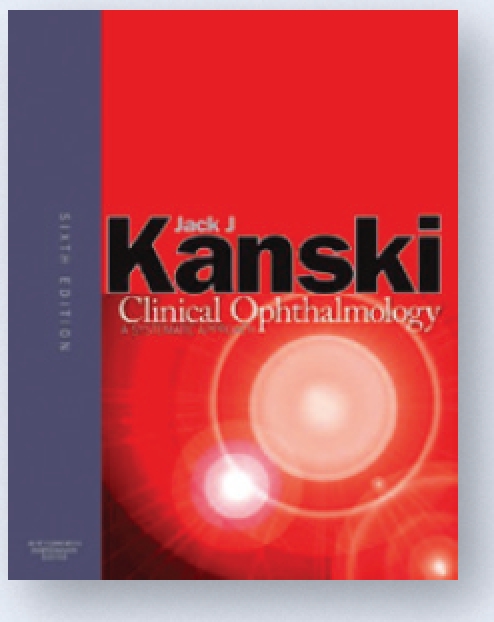

## Next issue

**Figure:**
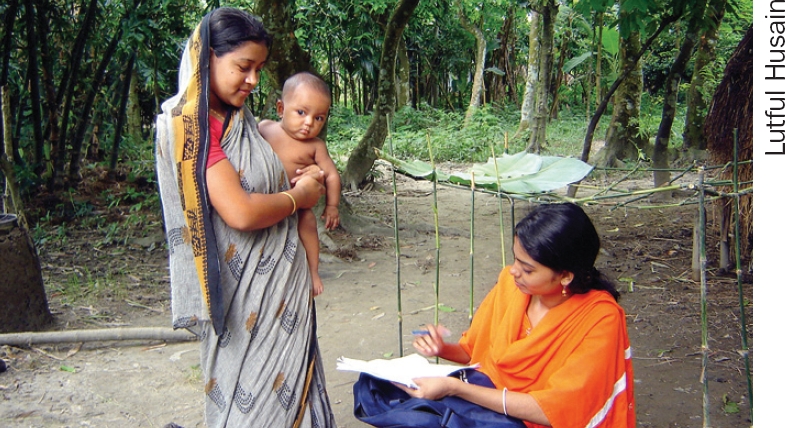
Lance Bellers/Sightsavers

The next issue of the *Community Eye Health Journal* will be on

**VISION 2020: why information matters**

